# Age-related changes and sex differences in ankle plantarflexion velocity

**DOI:** 10.1038/s41598-023-50275-1

**Published:** 2023-12-22

**Authors:** Atsuki Kanayama, Saki Yamamoto, Ryoga Ueba, Mio Kobayashi, Toshimitsu Ohmine, Akira Iwata

**Affiliations:** 1 Graduate School of Comprehensive Rehabilitation, Osaka Prefecture University, 3-7-30, Habikino, Habikino, Osaka 583-8555 Japan; 2 Graduate School of Rehabilitation Science, Osaka Metropolitan University, 3-7-30, Habikino, Habikino, Osaka 583-8555 Japan; 3 Division of Physical Therapy, Department of Rehabilitation Sciences, Faculty of Allied Health Sciences, Kansai University of Welfare Sciences, 3-11-1, Asahigaoka, Kashiwara, Osaka 582-0026 Japan

**Keywords:** Health care, Geriatrics

## Abstract

Ankle plantar flexors play a vital role in the mobility of older adults. The strength and velocity of plantarflexion are critical factors in determining walking speed. Despite reports on how age and sex affect plantarflexion strength, basic information regarding plantarflexion velocity is still lacking. This cross-sectional observational study investigated age-related changes and sex differences in plantarflexion velocity by comparing them with plantarflexion strength. A total of 550 healthy adults were classified into four age groups for each sex: Young (< 40 years old), Middle-aged (40–64 years old), Young-old (65–74 years old), and Older-old (≧ 75 years old). We measured plantarflexion velocity and strength in the long-sitting position using a gyroscope and a hand-held dynamometer, respectively. Two-way analysis of variance revealed no interaction between age and sex for either plantarflexion velocity or strength. Plantarflexion velocity exhibited a significant decline with aging, as did the plantarflexion strength. We found no significant sex differences in plantarflexion velocity in contrast to plantarflexion strength. The results indicated a significant decrease with age and no difference in plantarflexion velocity between males and females characteristic plantarflexion velocity. Understanding the characteristics of plantarflexion velocity could contribute to preventing a decline in mobility in older adults.

## Introduction

Maintaining mobility is critical for older adults to maintain independence in activities of daily living^1^. Walking speed is a commonly used indicator of mobility^2–4^ that markedly declines with age^5^. The primary reason for this decline is the changes in lower limb muscle function^6–9^.

The ankle plantar flexor muscles are one of the most vital lower limb functions that significantly affect the walking speed in older adults^10–12^. During gait, the plantar flexor muscles play a dominant role in push-off and contribute to forward propulsion^10,13^. The plantar flexors account for a considerable proportion (67%) of the total leg mechanical output during the propulsion phase of walking^14,15^. Several studies have demonstrated a significant correlation between walking speed and plantarflexion muscle strength in older adults^11,12,15–19^. In addition, a systematic review investigating plantarflexion concluded that there is substantial evidence supporting the relationship between plantarflexion isometric strength and walking speed^11^.

Recent studies have focused on lower limb movement velocity in addition to muscle strength^12,20^. Movement velocity represents the ability to move a joint as quickly as possible under low or no load conditions^12,20–24^. Previous study has suggested that movement velocity may have a more substantial impact on determining the mobility of older adults, as compared to muscle strength in knee extension function^23^. Additionally, the relationship between functional performance and maximum movement velocity strengthens as the external load is reduced and velocity increases^21^. In the context of ankle plantarflexion function, Arai et al. demonstrated a correlation between plantarflexion velocity during standing heel raise and walking speed in older adults^20^. Our previous study also showed that unloaded plantarflexion velocity in the sitting position is critical in determining walking speed, surpassing the importance of plantarflexion strength^12^. Consequently, it can be inferred that plantarflexion velocity is as crucial as or more critical than plantarflexion strength in determining walking speed among older adults.

The decline in plantarflexion strength and velocity with age is the primary cause of decreased walking speed in older adults. Therefore, understanding the characteristics of these functions is crucial when considering the maintenance and improvement of walking. Previous studies have reported age-related changes and sex differences in plantarflexion strength^25–30^, with a decrease of 34–42% from youth to old age^25,26^. In addition, males exhibit greater plantarflexion strength than females^27^. Although plantarflexion strength characteristics have been reported, no studies have investigated plantarflexion velocity in a wide range of subjects. Thus, the fundamental knowledge regarding age-related changes and sex differences in plantarflexion velocity remains unclear. This study aimed to clarify the age-related changes and sex-related differences in unloaded plantarflexion velocity by comparing them with isometric plantarflexion strength.

## Methods

### Participants

A total of 550 healthy adults (64.1 ± 19.0 years old, 205 males and 345 females) aged 18 to 91 from the community participated in this cross-sectional observational study. All participants were recruited by distributing leaflets at health-related events and community facilities and posting posters on bulletin boards at train stations and universities. Inclusion criteria for participation were as follows: (1) living in their own homes (not hospitalized or institutionalized), (2) absence of ankle joint disorders that impair plantarflexion movement and (3) ability to comprehend and comply with instructions. Participants were categorized into four age groups: young (< 40 years), middle-aged (40–64 years), young-old (65–74 years), and older-old (≧ 75 years). This study was conducted in accordance with the ethical principles outlined in the Declaration of Helsinki and approved by the Human Ethics Committee of Osaka Prefecture University (approval number: 2020-117, 2021-106, 2022-101). Informed consent was obtained from all participants for participation in the study and publication of information.

### Measurement data

Demographic data, including age, sex, height, body weight, body mass index (BMI), and ankle plantarflexion function, were collected from each participant. Ankle plantarflexion muscle strength and movement velocity were measured exclusively on the right side, and the maximum value was used for subsequent analyses. All measurements were performed by physical therapists on the same day.

**Figure 1:**
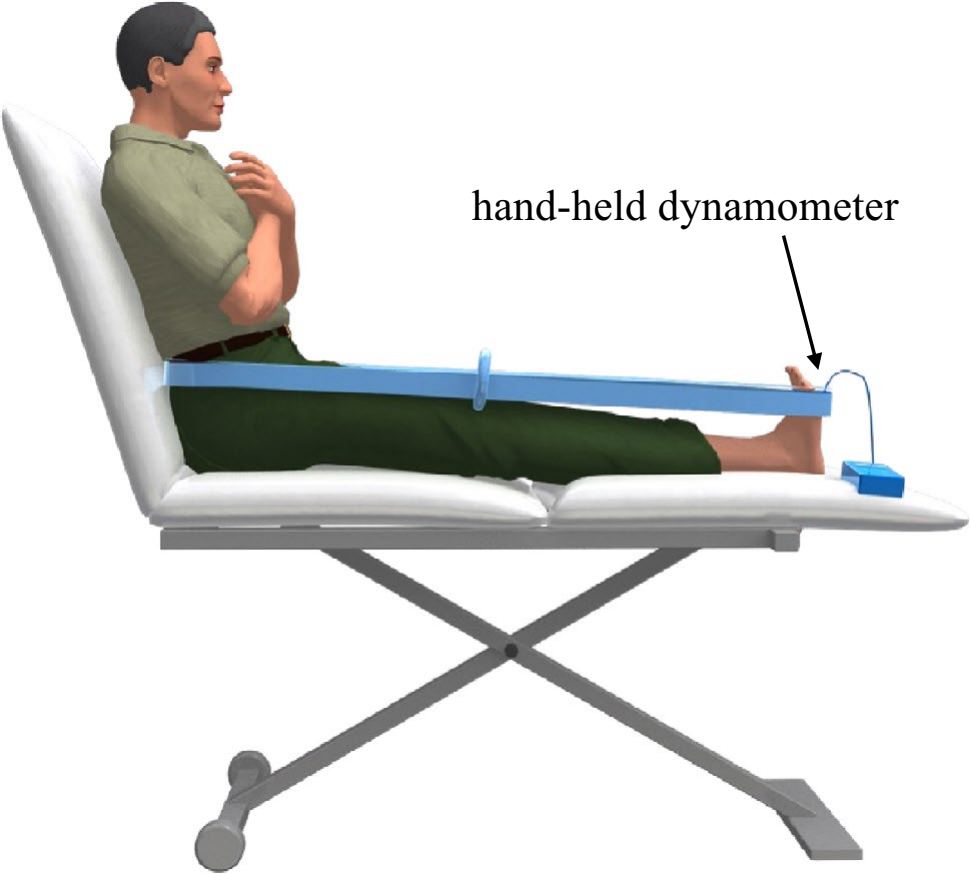
Measurement of ankle plantarflexion muscle strength. The participants sat on a bed with their backs against a backrest. The backrest was tilted backward by 30°. A hand-held dynamometer was attached to the sole, and the ankle joint was fixed in the neutral position.

The ankle plantarflexion muscle strength was assessed by measuring the isometric maximum muscle strength using a hand-held dynamometer (μTas F-100; ANIMA, Tokyo, Japan) (Fig. 1)^12^. The participants were seated in a long position on a bed with their arms crossed in front of their chests. The sensor pad of the hand-held dynamometer was positioned on the distal end of the metatarsus of the plantar foot, and the belt was firmly fastened to fix the ankle joint in the neutral position. The participants were instructed to exert maximum effort for 5 s during ankle plantarflexion. Two trials were conducted after one practice trial. Muscle strength data were normalized to body weight.

**Figure 2:**
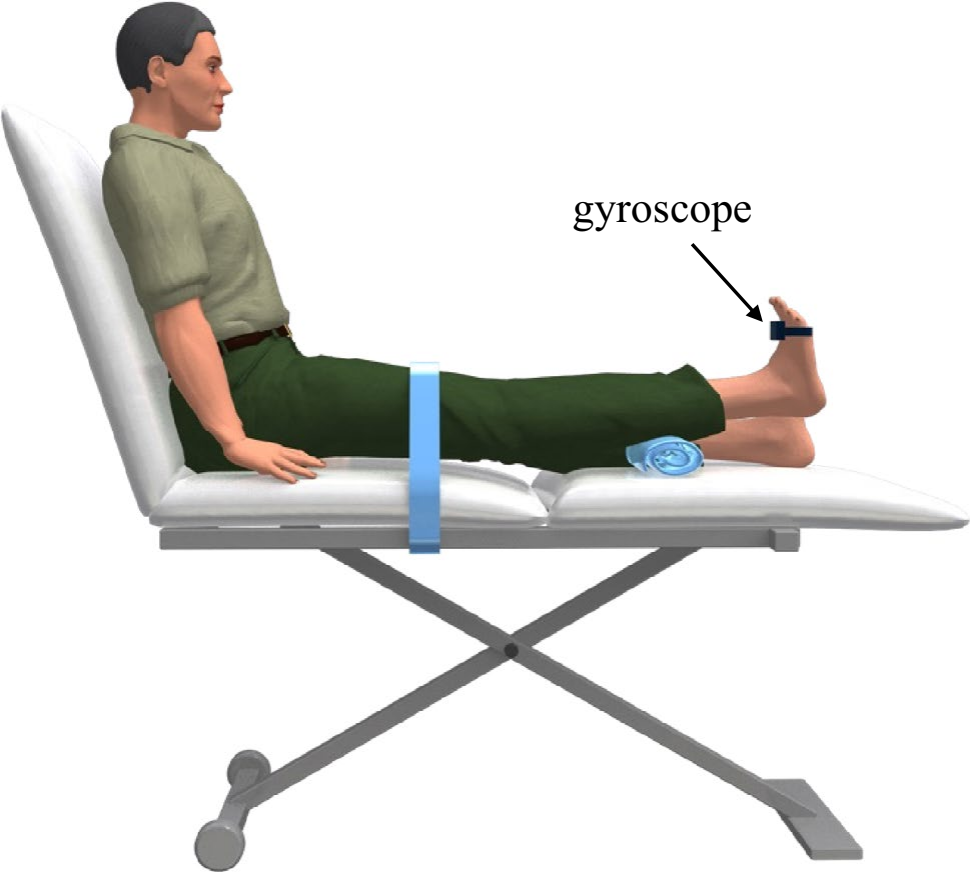
Measurement of ankle plantarflexion movement velocity. The participants sat on a bed with their backs against a backrest. The backrest was tilted backward by 30°. A gyroscope was attached to the dorsum of right foot.

The ankle plantarflexion movement velocity was assessed under unloaded conditions at the maximum angular velocity using a gyroscope (45 × 45 × 18 mm; MicroStone Corporation, Nagano, Japan) (Fig. 2)^12^. The gyroscope was attached to the distal end of the second metatarsal bone on the dorsal foot such that the axis of the gyroscope was aligned with the sagittal plane. The data frequency of the gyroscope was 200 Hz. The participants were seated in a long seated position on the bed with both hands on the floor supporting the body, and the lower limb was secured to the bed using a knee belt. The participants were instructed to execute ankle plantarflexion as quickly as possible within the ankle joint angle from maximum dorsiflexion to maximum plantarflexion. Five trials were conducted after two practice trials. Unfiltered data were used in the analysis. If a significant bimodal distribution was observed in the angular velocity data, the gyroscope was considered unsuitable for tracking foot movements and was excluded from the analysis.

### Statistical analysis

**Figure 3:**
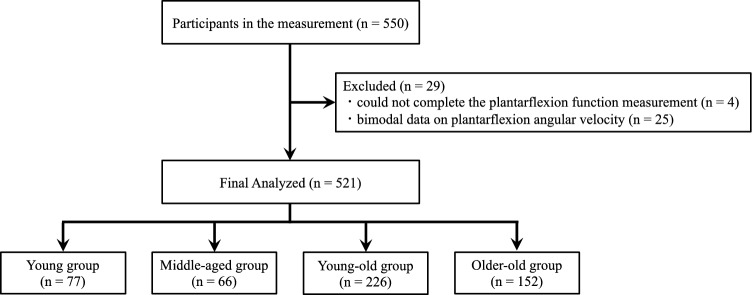
Flowchart of the participants and analyses.

Using G*Power 3.1 (Heinrich Heine University, Dusseldorf, Germany), we calculated the required sample size for a two-way analysis of variance (ANOVA) with an α error of 0.05, power (1 − β) of 0.80, and effect size (f) of 0.25. Consequently, the minimum sample size required for each group was determined to be 23. In statistical analysis, we utilized the maximum values of two measurements for ankle plantarflexion muscle strength and five for movement velocity. The data of four individuals who could not complete the plantarflexion function measurement and of 25 individuals with bimodal data on plantarflexion angular velocity were excluded from the analysis (Fig. 3). Initially, two-way ANOVA (age group × sex) was conducted to investigate the effects of age and sex on ankle plantarflexion muscle strength and movement velocity. If a significant interaction effect was detected, separate one-way ANOVAs, followed by Bonferroni post hoc tests, were performed to compare age groups and sex. If no interaction was observed, and there was a significant age effect, Bonferroni’s multiple comparison test was conducted to determine the location of the differences between the age groups. Simple regression analysis was performed to assess the contribution rate of plantarflexion muscle strength to plantarflexion movement velocity. All statistical analyses were conducted using SPSS statistics (version 28; IBM Corp, Armonk, NY, USA), with the significance level set at p < 0.05.

## Results

A total of 521 participants were included in the analysis. The young group comprised 77 individuals (40 males and 37 females); the middle-aged group, 66 individuals (32 males and 34 females); the young-old group, 226 individuals (55 males and 171 females); and the older-old group, 152 individuals (65 males and 87 females). All groups were well over the required sample size. The mean age and body size of the participants in each age group and sex are presented in Table 1.

**Table 1:** Characteristics of participants by age group and sex.

	Young (18–39 years)	Middle-aged (40–64 years)	Young-old (65–74 years)	Older-old (≦ 75 years)
Male (n = 192)				
n	40	32	55	65
Age (year)	21.7 (3.4)	55.8 (4.6)	70.1 (2.3)	79.4 (3.3)
Height (cm)	172.8 (5.4)	171.1 (6.3)	165.5 (6.2)	164.0 (5.8)
Body weight (kg)	64.5 (8.5)	70.9 (8.7)	66.5 (9.7)	61.8 (8.7)
BMI (kg/m^2^)	21.6 (2.4)	24.2 (2.2)	24.2 (2.9)	23.0 (3.0)
Female (n = 329)				
n	37	34	171	87
Age (year)	20.1 (1.3)	56.2 (4.3)	70.1 (2.7)	78.7 (3.2)
Height (cm)	159.9 (5.3)	157.0 (4.5)	153.0 (5.1)	151.4 (4.9)
Body weight (kg)	52.1 (6.1)	54.2 (9.2)	53.1 (8.0)	50.1 (8.6)
BMI (kg/m^2^)	20.4 (2.5)	22.1 (4.0)	22.7 (3.4)	21.8 (3.4)

Mean (standard deviation).

*BMI* body mass index.

**Figure 4:**
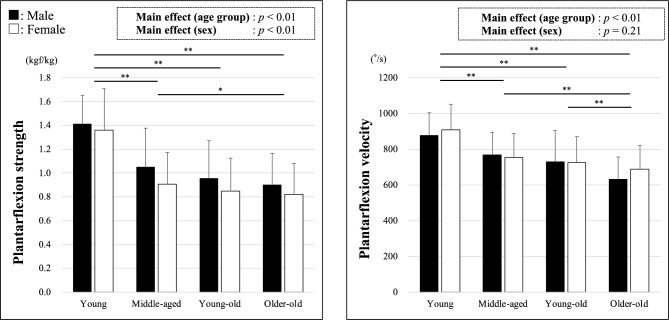
Age-related changes and sex differences of plantarflexion strength and velocity. The left-hand figure shows plantarflexion muscle strength, and the right-hand figure shows plantarflexion movement velocity. Bars represent the mean, and error bars represent the standard deviation. Black bars indicate data for males and white bars for females. *p < 0.05, **p < 0.01.

Table 2 and Fig. 4 show the measured plantarflexion strength and velocity across each age group and sex. Two-way ANOVA revealed no interaction between age group and sex for either plantarflexion strength or velocity. The main effects of age group and sex on plantarflexion strength were significant, with post hoc tests indicating significant differences between the young and middle-aged, young and young-old, young and older-old, and middle-aged and older-old age groups. Only a significant main effect of age was observed on plantarflexion velocity, with post hoc tests showing significant differences between the young and middle-aged, young and young-old, young and older-old, middle-aged and older-old, and young-old and older-old groups. Simple regression analysis showed that plantarflexion muscle strength could explain 16% of plantarflexion velocity (*β* = 0.401, p < 0.001, *R*^2^ = 0.159).

**Table 2:** The results of two-way analysis of variance.

Ankle plantarflexion	Mean (SD)					Interaction age × sex		Main effect of age		Main effect of sex	
	Young	Middle-aged	Young-old	Older-old	All groups	F-value	P-value	F-value	P-value	F-value	P-value
Muscle strength (kgf/kg)											
Male	1.41 (0.24)	1.05 (0.33)	0.95 (0.32)	0.90 (0.27)	1.05^§^ (0.35)	0.37	0.78	65.78	< 0.01	10.96	< 0.01
Female	1.36 (0.35)	0.90 (0.27)	0.85 (0.28)	0.82 (0.26)	0.90^§^ (0.32)						
Both sex	1.39 (0.30)	0.97* (0.30)	0.87* (0.29)	0.85*^,†^ (0.26)	0.96 (0.34)						
Movement velocity (°/s)											
Male	876.2 (128.8)	767.2 (128.2)	728.1 (175.1)	632.1 (124.1)	733.0 (166.9)	1.54	0.20	46.76	< 0.01	1.60	0.21
Female	909.2 (142.6)	754.5 (132.4)	724.2 (146.8)	688.0 (131.3)	738.6 (154.2)						
Both sex	892.1 (135.7)	760.7* (129.5)	725.2* (153.8)	664.1*^,†,‡^ (130.8)	736.5 (158.9)						

*SD* standard deviation.

*Significant difference compared with young (*p* < 0.05).

^†^Significant difference compared with middle-aged (*p* < 0.05).

^‡^Significant difference compared with young-old (*p* < 0.05).

^§^Significant difference between sex (*p* < 0.05).

## Discussion

The present study investigated age-related changes and sex differences in plantarflexion velocity and compared them with plantarflexion strength. Analysis of a sample of 521 individuals revealed that plantarflexion velocity decreased by approximately 26% from youth to old age, exhibiting an aging pattern resembling that of plantarflexion strength. Moreover, the sex difference in plantarflexion velocity was less than 1%, in contrast to the sex differences in plantarflexion strength. Therefore, plantarflexion velocity is characterized by a substantial decline with age and negligible sex disparities.

To the best of our knowledge, this is the first investigation to measure plantarflexion velocity during the unloaded condition across a wide range of subjects and demonstrate age-related changes and sex differences. This is a major strength of this study. Several factors may have influenced these age-related changes and sex-related differences. First, we discuss age-related changes. The present study detected a significant decrease in plantarflexion strength of approximately 39% between the young and older-old groups. Previous research has demonstrated that plantarflexion strength reduces by about 34–42% from youth to old age^25,26^. This reduction is due to age-related muscle mass loss (sarcopenia)^31,32^, alterations in muscle architecture^32–34^, and a decline in neuromuscular activation^35,36^. The observed rate of muscle weakness in the current study was consistent with previous investigations, indicating that we collected data from a standardized population.

First, this investigation revealed a significant 26% discrepancy between the younger and older groups regarding age-related changes in plantarflexion velocity. Muscle shortening velocity is a factor that contributes to age-related velocity changes^37^. The muscle shortening velocity of type II fibers was approximately three times greater than that of type I fibers^38^. Type II fibers show a more significant decrease than type I fibers, with age^39–43^, and the composition of skeletal muscle changes from youth to old age. Therefore, the shortening velocity of the skeletal muscle decreased systematically^44^. In addition, age-related modifications in neural function, such as an increase in antagonist muscle coactivation^36,45^ and a decrease in nerve conduction velocity^46^, result in reduced velocity. These age-related changes in both muscle and nerve functions led to a considerable reduction in plantarflexion velocity from younger to older people.

Second, sex-based differences showed divergent trends in strength and velocity. A significant sex difference in plantarflexion strength was observed, with males demonstrating approximately 14% greater values than females. This finding aligns with previous studies that reported greater plantarflexion strength in males than in females^27^. Conversely, no significant sex difference was observed in plantarflexion velocity, with a negligible difference of only 1% between the sexes. This is the first study to report no sex differences in joint movement velocity without external load. The absence of sex differences in plantarflexion velocity may be explained by two factors: ankle joint range of motion (ROM) and muscle–tendon complex (MTC) stiffness. ROM is the primary determinant of joint velocity because a greater extent of movement provides the limb with a longer acceleration duration^47^. Females have greater dorsiflexion ROM than males^48^, suggesting that they may have an advantage in generating velocity through ROM. However, a higher MTC stiffness is favorable for fast stretch–shortening cycle activities and high movement velocity^49^. It is widely recognized that males have greater muscle stiffness than females^49,50^. Therefore, males have the advantage of generating velocity through the MTC. The counterbalancing effects of male and female ankle joint ROM and MTC stiffness may explain the notable characteristic of no sex difference in plantarflexion velocity.

As described above, plantarflexion velocity exhibits unique characteristics that distinguish it from plantarflexion strength. Previous studies have shown a relatively weak correlation between unloaded velocity and isometric strength^12,22^. In line with these findings, the results of the single regression analysis in this study indicated that plantarflexion strength could account for only 16% of the variance in plantarflexion velocity. This suggests that movement velocity is an index independent of muscle strength. Additionally, lower limb movement velocity is a better predictor of walking speed than muscle strength^12,23^. Furthermore, the unloaded lower limb movement velocity can be easily measured, regardless of the location and subject. Thus, the usefulness and versatility of the unloaded movement velocity as a physical function indicator are significant.

This study had some limitations. Firstly, there was a significant discrepancy in the mean age between the young and middle-aged groups. Specifically, the male and female participants in the young group had mean ages of 21.7 ± 3.4 and 20.1 ± 1.3 years, respectively, while the male and female participants in the middle-aged group had mean ages of 55.8 ± 4.6 and 56.2 ± 4.3 years, respectively. This difference in average age is mainly due to the significantly lower number of participants in their 30 s and 40 s. Therefore, describing detailed aging patterns in these younger to middle-aged groups is not achievable in this study. The discernible age-related changes in this study are limited to comparing significant and substantial differences between young and older people. It is necessary to include additional age groups in their thirties and forties and more exhaustively scrutinize the decline from youth to middle age in future studies to address this gap. Secondly, this study only included healthy adults residing in the community, which could impact the data, especially for the older-old group. Many people aged 75 and above with low physical capabilities often require hospitalization or nursing home care, but this study did not collect data on such frail patients. Therefore, the results for the older-old group may be higher since the survey included many individuals with superior physical function in old age. While muscle strength typically declines significantly with old age^30^, the present study revealed a lower rate of decline in plantarflexion strength in older adults than in young and middle-aged adults. However, caution is necessary when interpreting the results as demographic characteristics of the study participants might affect the observed trends. Thirdly, the weak relationship between strength and velocity might be attributed to muscle strength measured using an isometric contraction. Previous research has demonstrated that age-related decline is more pronounced in isokinetic/isotonic strength compared to isometric strength^51,52^. One factor contributing to these differences in contraction types is that isokinetic/isotonic contraction requires a shorter time for muscle exertion to reach peak torque than isometric contraction^51^. Similar to isokinetic/isotonic contraction, measurement of movement velocity requires muscle exertion in a short time. Therefore, we can anticipate that isokinetic/isotonic muscle strength may exhibit a stronger association with movement velocity than isometric strength. Furthermore, future research should explore the relationship between muscle strength in different types of contractions and movement velocity.

## Conclusion

In conclusion, this study examined age-related changes and sex differences in unloaded plantarflexion velocity, a crucial determinant of mobility, by comparing it with plantarflexion strength. The findings revealed the following.

1. Both plantarflexion velocity and strength decrease significantly in the older groups compared to the young group.
2. A significant sex difference exists in plantarflexion strength but not in plantarflexion velocity.
3. Plantarflexion velocity is an independent index not solely dependent on plantarflexion strength.

Our findings show that older individuals experience an impactful decrease in muscle strength and movement velocity compared to their younger counterparts. Previous studies have unequivocally demonstrated the association between lower limb movement velocity and walking speed^12,20–24^. Therefore, the age-related changes observed in plantarflexion velocity are believed to considerably impact walking speed among older adults, emphasizing the critical importance of preventing a reduction in plantarflexion velocity. Additionally, since plantarflexion velocity in older adults is also related to static and dynamic balance function^20^, preventing age-related decline in plantarflexion velocity may effectively reduce the risk of falls. These findings are expected to be beneficial in the fields of rehabilitation as well as sports science and geriatrics.

## Data Availability

The datasets generated or analyzed in the current study are available from the corresponding author upon reasonable request.
